# LINC01503 in cancer: from molecular mechanisms to therapeutic implications

**DOI:** 10.1007/s10238-024-01383-3

**Published:** 2024-06-07

**Authors:** You Shuai, Haili Qian, Peng Yuan

**Affiliations:** 1 Department of Medical Oncology, National Cancer Centre/National Clinical Research Center for Cancer/Cancer Hospital, Chinese Academy of Medical Sciences and Peking Union Medical College, Beijing, 100021 China; 2 State Key Laboratory of Molecular Oncology, National Cancer Center/National Clinical Research Center for Cancer/Cancer Hospital, Chinese Academy of Medical Sciences and Peking Union Medical College, Beijing, 100021 China; 3 Department of VIP Medical Services, National Cancer Centre/National Clinical Research Center for Cancer/Cancer Hospital, Chinese Academy of Medical Sciences and Peking Union Medical College, Beijing, 100021 China

**Keywords:** Linc01503, Cancer, Mechanism, Clinical application

## Abstract

Long non-coding RNAs (lncRNAs) are fundamental agents that govern tumor growth and metastasis across a spectrum of cancer types. Linc01503 is a novel lncRNA situated on human chromosome 19, and it is intricately linked with the pathogenesis of multiple human cancers, underscoring its substantial role and significance in cancer development. It has been recognized as a pivotal contributor to inducing malignant behaviors in lung cancer, gastric cancer, colorectal cancer, cholangiocarcinoma, liver cancer and pancreatic cancer, among others. The dysregulation of linc01503 has been shown to strongly associate with advanced clinicopathological factors and foretell an unfavorable prognosis, indicating its prospective clinical significance as a valuable biomarker and therapeutic target for individuals with cancer. The primary objective of the current work is to present the intricate molecular pathways governed by linc01503 and its profound clinical relevance in the context of carcinogenesis. We also focus on the future prospects of linc01503-based clinical application. This will help us to better understand the regulatory mechanism of carcinogenesis and provide new ideas for precision molecular medicine.

## Introduction

Cancer is a major public health problem worldwide [1]. It is a complex pathological event that can be initiated by a multitude of driving forces and influences, encompassing gene dysregulation, epigenetic modifications, environmental factors, etc. [2]. Delays in the diagnosis and treatment may culminate in an escalation of advanced-stage disease and higher mortality rates, which calls for identification of novel biomarkers [2]. Therefore, it is imperative to elucidate the mechanistic networks underlying tumorigenesis and progression to enhance cancer diagnosis and treatment. The recent identification of genetic alterations and dysregulation of critical factors involved in carcinogenesis offers new opportunities for precision molecular medicine, with long non-coding RNAs (lncRNAs) as promising biomarkers.

**Fig. 1:**
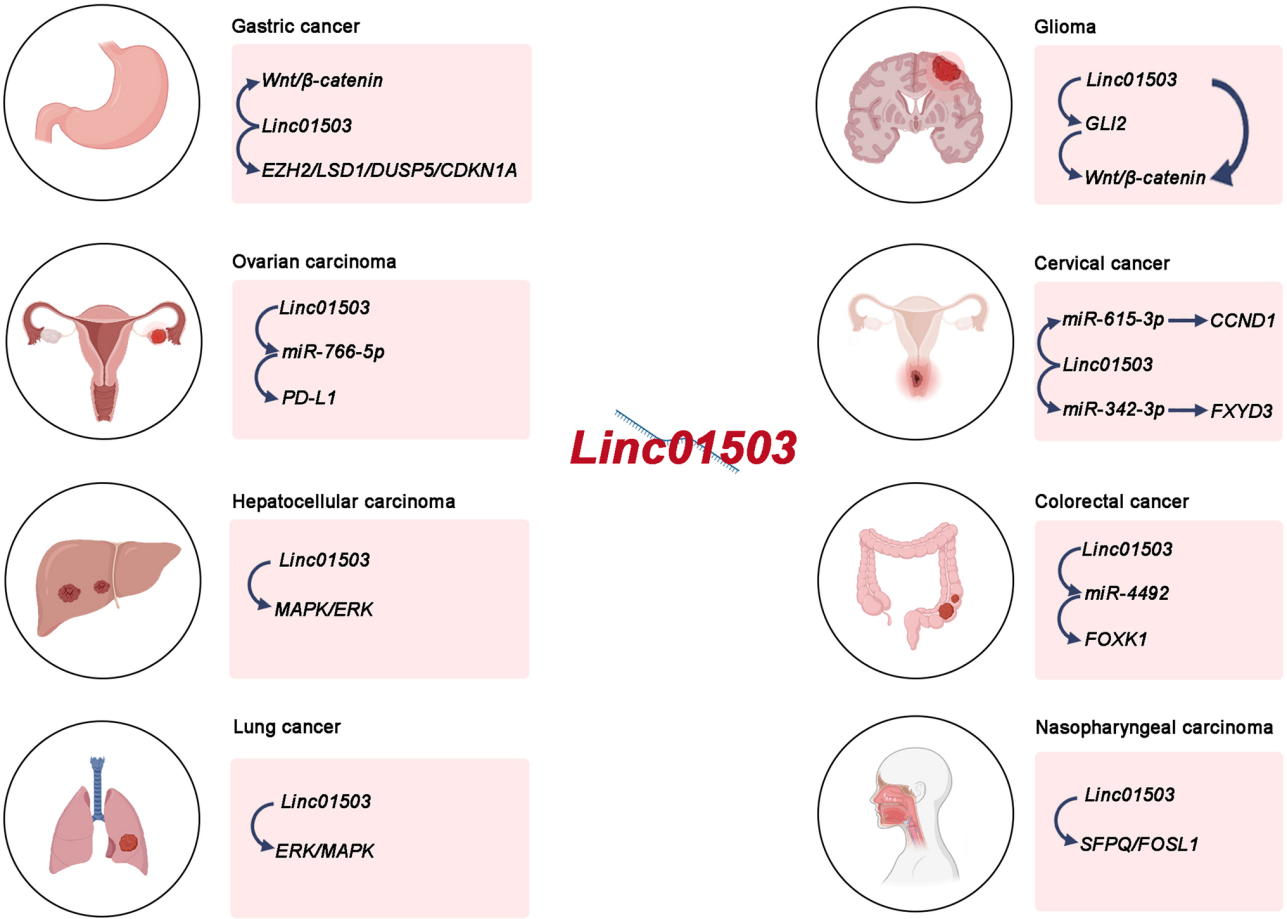
The molecular mechanisms of linc01503 in various human cancers

**Fig. 2:**
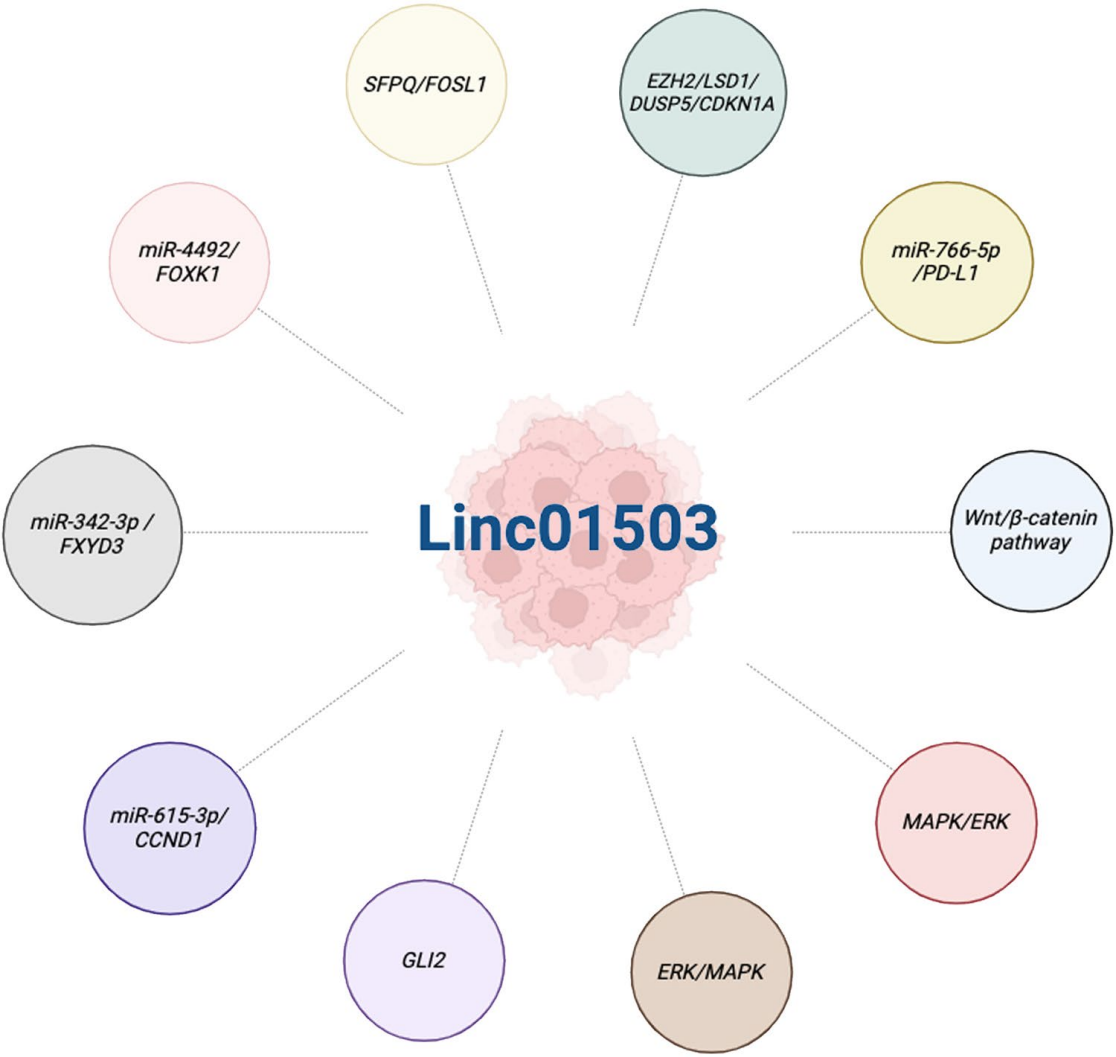
Regulatory mechanisms associated with linc01503

**Fig. 3:**
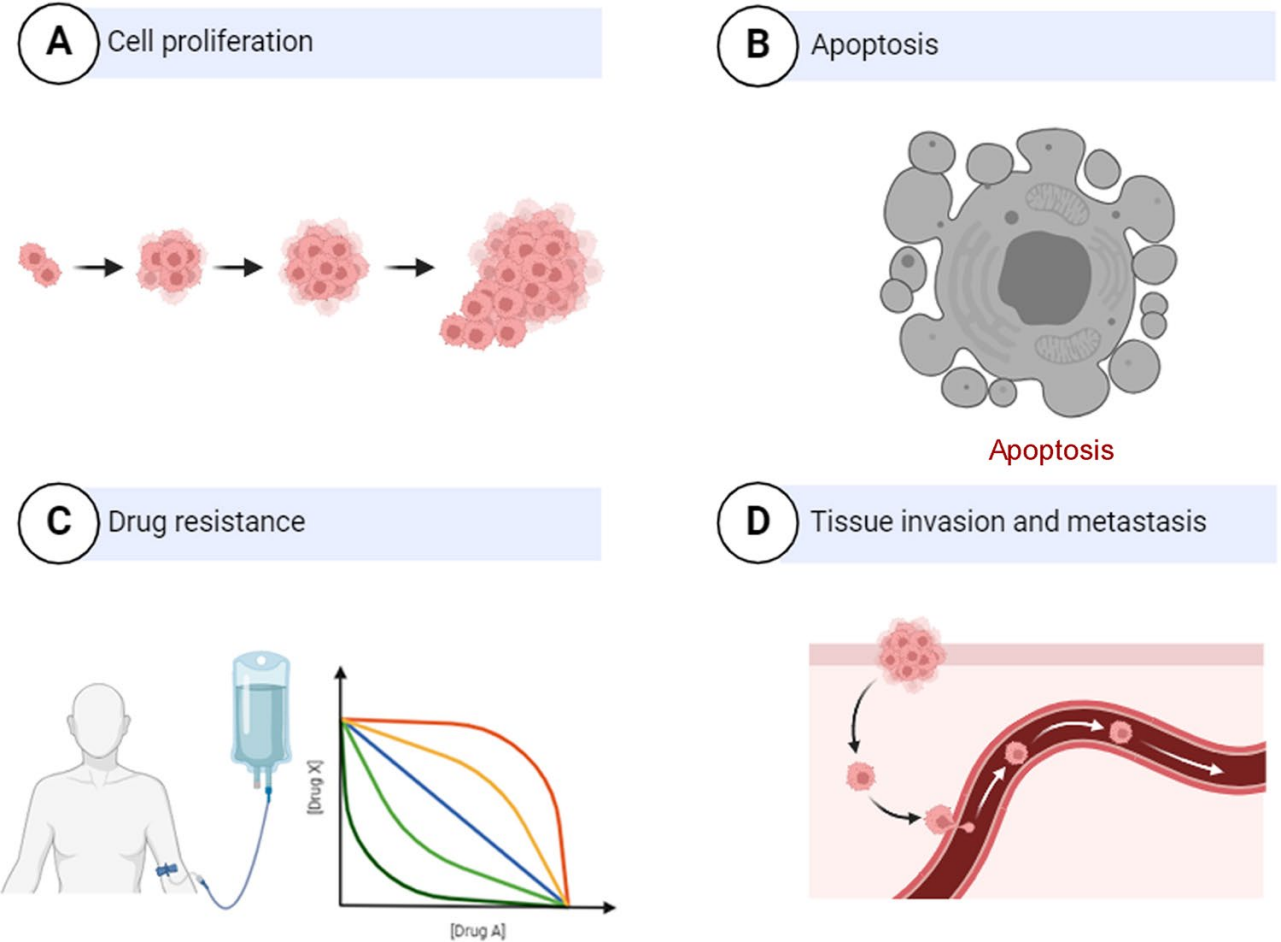
The biological function of linc01503

LncRNAs, consisting of more than 200 nucleotides, govern gene expression and contribute to various biological processes through a range of pathways and molecular mechanisms [3]. Recent research indicates that lncRNAs have significant implications in various cancer types. Their involvements encompass sequestering microRNAs, engaging with RNA-binding proteins, modulating gene transcription, influencing alternative splicing, and affecting protein translation [4, 5]. Linc01503 is a novel lncRNA located in human chromosome 19 and closely correlated with human cancer development, including lung cancer, gastric cancer, colorectal cancer, cholangiocarcinoma, liver cancer, etc. [6–10] (Fig. 1). For instance, it was initially identified to be an oncogenic regulator in aggressive squamous cell carcinoma (SCC) [11]. Our research team firstly investigated the upstream regulator essential for aberrant expression of linc01503 in gastric cancer (GC) [7]. The transcription factor early growth response protein 1(EGR1) activated the transcription of linc01503 in GC [7]. Further, linc01503 was identified to silence the expression of cyclin‐dependent kinase inhibitor 1A (CDKN1A) and dual‐specificity phosphatase 5(DUSP5) through the interaction with lysine (K)‐specific demethylase 1A (LSD1), thus facilitating GC progression and enhancer of zeste 2 (EZH2) [7]. Of note, the dysregulation of linc01503 exhibited close association with clinical factors and malignant behaviors in various malignancies, affecting the prognosis of cancer patients [7, 8, 11]. These interesting observations highlight the critical involvement of linc01503 in various types of cancers (Figs. 2, 3).

In this review, the primary objective is to present the intricate molecular pathways governed by linc01503 and its profound clinical relevance in the context of carcinogenesis (Table 1). We also focus on the future prospects of linc01503-based clinical application (Table 2). This in-depth summary toward better understanding linc01503 involvement in human cancers may inspire the identification of novel mechanistic networks and effective biomarkers for cancer therapeutics.

## The underlying mechanism and clinical potentials of linc01503 in malignant diseases

### Gastric cancer

GC, a widespread malignancy globally, carries a significant risk of metastasis and recurrence [12]. It holds the fifth position in global incidence and ranks third in mortality [13]. Mounting evidence has unveiled the significance of lncRNAs as crucial regulators in a range of cancers, including GC [14]. Recently, linc01503 has been found in several studies to be strikingly upregulated in GC tissue samples [6]. In GC patients, clinicopathological factors, including TNM stage, lymph node metastasis, and age, were assessed in tissue specimens with varying linc01503 expression levels, in comparison with those from normal gastric tissues [6]. This interesting research directed by Ding et al. indicated a strong association between increased linc01503 level and advanced pathological stage of GC patients, with a statistically significant *p* value of 0.0163 [6]. Furthermore, Ding et al. detected the expression of linc01503 across distinct subgroups of GC and aimed to determine whether linc01503 demonstrated specific associations with particular subtypes [6]. It was elucidated that linc01503 expression notably rose in poorly and moderately differentiated GC versus the well-differentiated type [6]. It was notable that GC cases exhibiting elevated linc01503 levels correlated with reduced survival rates, typically averaging around 18.23 months [6]. These observations highlighted that linc01503 may be a promising marker for GC diagnosis and prognostic assessment.

Further research showed that linc01503 accelerated the malignant progression of GC by positively inducing the Wnt/*β*-catenin pathway, revealing its potential as a therapeutic target for patients with GC [6]. Our previous research enriched the understanding of mechanistic model induced by linc01503 in GC tumorigenesis [7]. Silencing of linc01503 could lead to the activation of apoptotic capacities of GC cells [7]. Mechanistically, EGR1 critically activated the transcription of linc01503 [7]. Furthermore, linc01503 could interact with LSD1 and EZH2, thereby silencing CDKN1A and DUSP5 expression [7]. These findings illuminated that linc01503 could act as an oncogene in GC progression and may be a promising target for GC therapeutics. However, larger tumor cases and longer follow-up visits are in an urgent need. Further, deep mechanistic research are required to enrich the understanding of linc01503 in GC carcinogenesis, especially at the post-transcriptional level.

### Ovarian carcinoma

Ovarian carcinoma (OCa), a highly fatal malignancy of the female reproductive system, stands as the second most prevalent gynecological cancer globally, accounting for about one-third of all such malignancies [15]. There is growing evidence pinpointing the significance of lncRNAs in mediating OCa chemoresistance [16]. Interestingly, Li et al. elucidated the involvement of linc01503 in OCa resistance to carboplatin (CBP) [17]. Li et al. demonstrated that linc01503 was obviously increased in CBP-resistant OCa cells. The transcription of linc01503 in CBP-resistant OCa cells was stimulated by GATA-binding protein 1 (GATA1) [17]. Notably, linc01503 impairment could lead to decreased carboplatin (CBP) resistance in OCa cells [17]. Li et al. verified that linc01503 targets miR-766-5p, which showed reduced levels in CBP-resistant cells [17]. Furthermore, linc01503 boosted PD-L1 levels through modulating miR-766-5p levels. Elevated PD-L1 levels reversed the attenuated effect of linc01503 inhibition on CBP resistance in OCa cells [17]. These findings suggested the significance of the GATA1-activated linc01503 mechanism network in mediating CBP resistance in OCa, illuminating a prospective target for enhancing the effectiveness of OCa chemotherapy [17].

However, the study performed by Li et al. mainly focused on the role of linc01503 in conferring resistance to CBP in OCa cells and lacked the validation assays at clinical level and animal level. The correlations between linc01503 level and clinical–pathological features of OCa patients were also warranted. Besides, the in vivo effects and other mechanistic models of linc01503 remain to be elucidated in future investigations (Fig. 3).

### Hepatocellular carcinoma

Hepatocellular carcinoma (HCC) ranks as the second leading cause of tumor-related deaths globally, characterized by notably high mortality rates, with approximately 800,000 new cases annually [18]. Emerging evidence has illuminated the critical significance of lncRNAs in diverse malignancies, particularly in the pathogenesis of HCC [19]. A recent study directed by Wang et al. determined the role of linc01503 in HCC progression [20]. It was found that linc01503 was substantially increased in HCC and showed close association with tumor grade, TNM stage and T classification [20]. The elevated linc01503 level in HCC corresponded to a more severe progression in stages, grades, and T classifications compared to lower levels [20]. Patients with elevated level of linc01503 in HCC exhibited significantly lower overall survival rates compared to those with lower expression [20]. Multivariate and univariate Cox regression analyses showed that linc01503 level independently predicted overall survival in HCC patients (HR 1.26,* P* = 0.046) [20]. These observations revealed the typical expression pattern of linc01503 and its clinical significance in HCC patients, highlighting its potential as an effective biomarker for HCC detection and prognostic assessments.

Functional experiments uncovered that linc01503 knockdown substantially inhibits the proliferation of HCC cells and induced apoptosis [20]. In terms of mechanisms, KEGG analysis revealed that the altered genes mediated by linc01503 exhibited close association with MAPK signaling pathway [20]. Overexpression of linc01503 increased p-ERK levels, while silencing of linc01503 exhibited the opposite effects in HCC cells [20]. Linc01503 was identified to activate the MAPK/ERK signaling pathway, and the inhibition of this pathway could mitigate the pro-tumor effects of linc01503 [20]. These findings suggested that linc01503 could promote HCC proliferation through regulating MAPK/ERK signaling pathway, highlighting the molecular axis of linc01503/MAPK/ERK as promising biomarkers for HCC detection and treatment [20]. Yet, the precise binding sites governing the interaction between LINC01503 and the MAPK/ERK pathway remain elusive. Therefore, conducting further in-depth research is imperative to fully uncover this molecular cross-talk (Table 1).

**Table 1:** Linc01503 in human cancers

Cancer	Expression	Functional	Related gene	Role	References
Gastric cancer	Upregulated	Proliferation, migration, invasion	Wnt/*β*-catenin pathway	Oncogene	[6]
	Upregulated	Proliferation, apoptosis	EZH2/LSD1/DUSP5/CDKN1A	Oncogene	[7]
Ovarian carcinoma	Upregulated	Proliferation, migration, invasion, apoptosis, drug resistance	miR-766-5p/PD-L1	Oncogene	[17]
Hepatocellular carcinoma	Upregulated	Proliferation, apoptosis	MAPK/ERK	Oncogene	[20]
Lung cancer	Upregulated	Proliferation, apoptosis	ERK/MAPK pathway	Oncogene	[9]
Glioma	Upregulated	Proliferation, migration, invasion, apoptosis	Wnt/*β*-catenin pathway	Oncogene	[22]
	–	Glioblastoma stem cell properties	GLI2, Wnt/*β*-catenin pathway	–	[23]
Cervical cancer	Upregulated	Proliferation, invasion	miR-615-3p/CCND1	Oncogene	[24]
	Upregulated	Proliferation, apoptosis	miR-342-3p /FXYD3	Oncogene	[25]
Colorectal cancer	Upregulated	Proliferation, invasion	miR-4492/FOXK1	Oncogene	[8]
Cholangiocarcinoma	Upregulated	Proliferation, migration, invasion, EMT	–	Oncogene	[10]
Nasopharyngeal carcinoma	Upregulated	Proliferation, migration, invasion	SFPQ/FOSL1	Oncogene	[28]

### Lung cancer

Lung cancer ranks among the leading causes of cancer-related fatalities globally. Non-small cell lung cancer (NSCLC), the predominant form of lung malignancy, represents roughly 85% of all lung cancer diagnoses [21]. The evolution and progression of NSCLC involve a multifaceted process influenced by numerous genes, yet its intricate molecular pathogenesis remains inadequately understood. The dysregulation of lncRNAs in NSCLC could potentially accelerate lung cancer progression. Zhang et al. determined the significantly elevated expression of linc01503 in NSCLC tissue samples [9]. Functionally, knockdown of linc01503 attenuated the proliferative capacity of NSCLC cells and increased the apoptosis rate of NSCLC cells [9]. A positive regulatory correlation was observed between c-MYC and linc01503 [9]. Further, linc01503 knockdown could obviously inhibit the expression of phosphorylated ERK1/2 (p-ERK1/2) and p-MAPK/ERK kinase (MEK), the molecular markers in the ERK/MAPK signaling pathway [9]. These findings uncovered the function of linc01503/ERK/MAPK network in advancing NSCLC progression, offering novel insights into molecular diagnostics and therapeutics for patients with NSCLC.

It is worth noting that massive efforts should be done to clarify the clinical value and deep mechanism of linc01503 in future work. The relationship between linc01503 expression and the clinical–pathological characteristics and survival prognosis of lung cancer patients holds significant importance. However, this area remains largely unexplored. Therefore, it is imperative to prioritize and thoroughly investigate this aspect. Besides, the direct integration of C-Myc into the linc01503 promoter requires chromatin immunoprecipitation (ChIP) experiments for verification. More endeavors should target uncovering other regulatory pathways and investigating the clinical relevance of linc01503 in lung cancer in forthcoming research.

### Glioma

Glioma, a prevalent type of primary intracranial malignancy, is characterized by rapid cellular proliferation and the formation of new blood vessels. The primary contributors to the bleak prognosis are rapid proliferation and swift metastasis in the early stages. Hence, there is an urgent need for significant efforts to identify potential biomarkers for glioma patients. Recently, the significant elevation of linc01503 has been consistently observed in glioma tissues and cells [22]. Glioma patients with larger size and advanced WHO grade exhibited higher linc01503 expression [22]. Moreover, higher linc01503 level was correlated with worse disease-free survival and overall survival [22]. Linc01503 was determined to act as an unfavorable prognostic factor for patients with glioma [22].

Functional assays further demonstrated that knockdown of linc01503 could suppress cell growth, migration and invasion, while promoting apoptosis. Further, linc01503 has been implicated in facilitating the activation of the Wnt/*β*-catenin signaling pathway, thereby contributing to the glioma progression [22]. Another study directed by Wei et al. revealed that linc01503 participated into the mediation of multiple GSC markers [23]. Moreover, the transcript of linc01503, ENST00000444125, was identified to reduce GLI2 ubiquitination and partially attenuate FBXW1 induced GLI2 ubiquitination [23]. These findings indicated that the major functional transcript of linc01503 could enhance the GSC properties of GBM cells via reducing FBXW1-mediated proteasomal degradation of GLI2 [23]. However, the in vivo effects and deep understanding concerning the mechanistic model of linc01503 still determine to be clarified in future research (Table 2).

**Table 2:** Clinical significance of linc01503 in various tumors

Type of cancer	Associated clinical features	References
Cholangiocarcinoma	Lymph node, metastasis	[10]
Hepatocellular carcinoma	Tumor volume, metastasis, TNM stage	[20]
Glioma	KPS, tumor size, WHO grade, overall survival, disease-free survival	[22]
Glioma	Overall survival, progression-free survival	[23]
Cervical cancer	FIGO stage, lymph-node metastasis, depth of invasion	[24]
Nasopharyngeal carcinoma	Overall survival, disease-free survival, distant metastasis-free survival	[28]

### Cervical cancer

Cervical cancer stands as the second most frequent malignancy in females, significantly impacting cancer-related mortality. Despite extensive research into its pathogenesis, the prognosis for cervical cancer patients continues to be bleak. A striking study reported by Feng et al. uncovered the biological role and molecular mechanism of linc01503 in cervical cancer. It was found that linc01503 was highly expressed in cervical cancer tissues [24]. Increased expression of linc01503 was associated with the advancement of cervical cancer, characterized by higher FIGO staging, augmented tumor cell metastasis to lymph nodes, and increased infiltration into deeper cervical tissues [24]. Moreover, the inhibition of linc01503 could markedly impair tumor cell proliferation and invasion through interacting with miR-615-3p/CCND1 in cervical cancer [24]. These findings highlight the therapeutic potential of targeting the LINC01503/miR-615-3p/CCND1 axis in cervical cancer therapeutics.

Another study directed by Feng et al. enriched the regulatory mechanism of linc01503 at the post-transcriptional level [25]. It was revealed that linc01503 acted as the endogenous sponge for miR-342-3p and positively regulated FXYD3 expression in cervical cancer, thereby driving cervical cancer progression and providing prospective targets for patients with cervical cancer [25]. However, more samples should be collected and included in the study to investigate the expression levels of linc01503 in tissue specimens and its correlation with clinical pathological features. Furthermore, it is crucial to prioritize patient follow-ups, meticulously recording information pertaining to changes in their medical condition. This comprehensive approach will help clarify the correlation between linc01503 expression levels and patients’ survival prognosis. Currently, investigations into the role of linc01503 in cervical cancer predominantly focus on post-transcriptional processes. However, it is noteworthy to notice the potential dual localization of linc01503 within both the cytoplasm and nucleus. Hence, it is imperative to delve deeper into elucidating additional molecular mechanisms orchestrated by linc01503, particularly its regulatory actions at the transcriptional level.

### Colorectal cancer

Colorectal cancer (CRC) ranks as the third most prevalent cancer globally, accounting for an estimated 1.3 million new cases and causing approximately 690,000 deaths annually. Despite significant advancements in CRC treatment over recent decades, the prognosis, notably for advanced-stage tumors with distant metastasis, continues to pose a challenge, with outcomes remaining less than satisfactory. Mounting evidence suggests a strong correlation between lncRNAs and the advancement of CRC. An interesting study reported by Lu et al. elucidated the involvement of linc01503 in CRC progression, providing novel insights into lncRNA-based molecular diagnosis and therapy.

Linc01503 was identified to be highly expressed in CRC tissue samples and cell lines [8]. Functional assays found that linc01503 knockdown could obviously inhibit CTC cell proliferation and invasion [8]. In contrast, the elevation of linc01503 displayed the opposite effects. Lu et al. uncovered that linc01503 acts as a competitive endogenous RNA, sequestering microRNA (miR)-4492, consequently modulating the expression of forkhead box K1 (FOXK1) within CRC cells [8]. Upregulation of miR-4492 mimics led to the inhibition of FOXK1 expression, while simultaneous overexpression of linc01503 counteracted this effect [8]. Moreover, the restoration of FOXK1 reversed the suppressive impact of linc01503 depletion on CRC cell proliferation and invasion [8]. Overall, linc01503 functioned as a ceRNA to reduce miR-4492/FOXK1 complex, thereby facilitating CRC progression [8]. These findings revealed that targeting of linc01503//miR-4492/FOXK1 axis may serve as an effective target for CRC patients.

However, current studies concentrate on the impact of linc01503 on CRC cell proliferation and invasion and uncovered its molecular mechanism at the post-transcriptional level. The exploration and verification of in vivo assays are required to uncover the role of linc01503 in vivo. Besides, cell death has been identified to play a significant role in carcinogenesis. Further investigations are warranted to detect the effects of linc01503 on CRC cell death, such as apoptosis and pyroptosis. It may be of great interest to uncover deep mechanism of linc01503 in CRC progression. Importantly, clinical specimens should be involved to reveal the expression pattern of linc01503 and its potential correlation with clinical features, as well as prognostic survival.

### Cholangiocarcinoma

Cholangiocarcinoma, the second most prevalent malignancy in the hepatobiliary system, originates from the epithelial cells of the biliary tract. Recent years have witnessed divergent incidence rates across regions and genders, yet a consistent annual upsurge in global prevalence is evident. Providing an elucidation of the molecular mechanisms underlying the occurrence and progression of cholangiocarcinoma holds significant importance. A recent study reported by An et al. determined the function of linc01503 in cholangiocarcinoma. The expression level of linc01503 exhibited a significant increase in cholangiocarcinoma tissues compared to adjacent tissues [10]. Moreover, the elevated expression of linc01503 showed correlation with lymph node metastasis, highlighting the crucial involvement of linc01503 in cholangiocarcinoma [10].

In contrast to normal bile duct cells (HIBEC), cholangiocarcinoma cells (RBE, QBC939) displayed elevated expression levels of linc01503 [10]. The inhibition of linc01503 could dramatically suppress cell proliferation and invasion in cholangiocarcinoma [10]. It was notable that linc01503 knockdown could lead to the obvious increase in E-cadherin and significant decrease in N-cadherin and Vimentin at the protein level [10]. These observations demonstrated that linc01503 was involved in mediating the epithelial-mesenchymal transition (EMT) process, thus contributing to cell migration and invasion [10]. This research directed by An et al. offered a foundational premise for investigating the mechanism associated with linc01503 and revealed the potential of linc01503 as prospective biomarkers for diagnosis and treatment.

Nevertheless, the current knowledge regarding linc01503 in cholangiocarcinoma remains significantly insufficient. In future research endeavors, elucidating the precise cellular localization of LINC01503 is imperative to unravel its broader functions and delve deeper into its molecular mechanisms. Although An et al.’s research revealed the regulatory role of LINC01503 in the EMT process, a targeted emphasis on elucidating the underlying mechanisms requires dedicated attention in future investigations.

### Nasopharyngeal carcinoma

Nasopharyngeal carcinoma (NPC) represents an epithelial malignancy arising from the mucosal lining within the nasopharyngeal cavity [26]. NPC exhibits a notably elevated incidence within Southeast Asia, notably prevalent in China, where it accounts for almost 40% of newly diagnosed cases worldwide [27]. A majority exceeding 70% of NPC cases are diagnosed at an advanced stage, where local relapse and distant metastasis stand as predominant factors contributing to NPC-related mortality [27]. Therefore, the identification of key biomarkers and comprehensive elucidation of their mechanistic roles in NPC recurrence and metastasis hold paramount significance in paving the way for tailored therapeutic interventions for NPC patients.

A striking study directed by He et al. furthered the depth of understanding regarding the regulatory mechanisms of linc01503 in NPC [28]. It was observed that linc01503 was obviously overexpressed in NPC [28]. Patients with higher levels of linc01503 demonstrated inferior rates in overall, disease-free, and distant metastasis-free survival in contrast to individuals exhibiting lower levels of linc01503 [28]. Further, linc01503 was determined to promote NPC cell proliferation, migration, and invasion in vitro and to facilitate tumor growth and metastasis in vivo [28]. Importantly, linc01503 recruited the splicing factor proline- and glutamine-rich (SFPQ) to activate the transcription of Fos like 1 (FOSL1) [28]. Furthermore, the overexpression of FOSL1 counteracted the suppressive impact caused by the knockdown of linc01503 on the progression of NPC [28]. The activation of androgen receptor (AR)-mediated transcription resulted in the upregulation of linc01503, consequently promoting AR ligand-dependent cell growth and invasion in NPC [28]. The discovery of this AR/linc01503/SFPQ/FOSL1 pathway introduces a novel prognostic biomarker and therapeutic target for NPC patients.

The regulatory mechanisms governing aberrant gene expression are notably intricate. He et al. elucidated a close correlation between elevated expression levels of linc01503 and the transcription factor AR [28]. Further investigations are warranted to explore the potential interaction between the heightened expression of linc01503 and other pivotal transcription factors [28]. Additionally, recent research has unveiled the potential impact of abnormal m6A modifications on the dysregulation of lncRNAs. Examining the intricate relationship between m6A modifications and the regulatory role of linc01503 stands as a compelling and imperative avenue for future scholarly investigation.

## The utility of linc01503 as prospective biomarkers or targets in cancer clinics

Evidence has highlighted the considerable potential of lncRNAs as emerging biomarkers in scientific investigations. LncRNAs are discernible in various biological specimens, including tissue samples, saliva, or plasma, exhibiting cell-specific or stage-specific expression patterns, which partly contribute to the potential of lncRNAs as promising biomarkers. These characteristics can partly account for the possible application of lncRNAs as prospective biomarkers. Our previous research illuminated that linc01503 was dramatically increased in GC tissue samples [29]. Its amplification was positively associated with pathological stages and unfavorable prognosis for patients with GC, indicating its potential as a novel biomarker for GC diagnosis and prognostic assessments. Furthermore, the oncogenic role of linc01503 in facilitating the proliferation and metastasis of GC cells enabled it a promising biomarker. Another study by Feng et al. detected the significant increase of linc01503 in tissue samples from cervical cancer patients, which suggested that the elevated expression of linc01503 can distinguish patients with cervical cancer from healthy individuals [24]. Patients with higher level of linc01503 conferred poorer clinical staging. Further, linc01503 was demonstrated to promote cervical cancer cell proliferation and invasion and may become a prospective noninvasive biomarker for cervical cancer patients [24]. As for HCC [20], linc01503 could promote HCC proliferation through mediating MAPK/ERK signaling pathway. Elevated levels of linc01503 in HCC were linked to more severe progression and lower survival rates, establishing it as a valuable biomarker for cancer screening and prognostic monitoring [20]. Moreover, linc01503 was identified to be highly expressed in glioma tissue samples and acted as an oncogene in glioma progression [22]. Higher expression level of linc01503 predicted larger tumor size, more advanced SHO grade and worse survival condition, highlighting its utility as an effective biomarker for prognostic prediction [22].

In recent years, attention has increasingly shifted toward investigating lncRNAs as potential therapeutic targets. Interestingly, linc01503 has garnered significant attention for their roles in tumor development and regulation. The effective techniques of gene knockdown or overexpression may shed new light on the targeting of lncRNAs. For oncogenic lncRNAs, CRISPR-Cas13a, a flexible platform, was applied to implement programmable knockdown with reduced off-target impacts. For tumor-suppressive lncRNAs, the overexpression vectors could promote the aberrant expression of lncRNAs. Interestingly, linc01503 has garnered significant attention for their roles in tumor development and regulation. Zhang et al. reported the elevated level of linc01503 in NSCLC tissue samples and linc01503 suppressed the expression of p-ERK1/2 and p-MAPK/ERK to play oncogenic role in lung cancer [9]. Targeting of linc01503/p-MAPK/ERK axis may bring much benefits for patients with lung cancer [9]. In cervical cancer, increased expression of linc01503 was identified to be closely associated with cervical cancer progression, characterized by higher FIGO staging, augmented tumor cell metastasis to lymph nodes, and increased infiltration into deeper cervical tissues [24]. Linc01503 acted as an oncogenic regulator through inducing miR-615-3p/CCND1 in cervical cancer [24]. Another study investigated by Lu et al. revealed that linc01503 functioned as a ceRNA to reduce miR-4492/FOXK1 complex, thereby facilitating CRC progression [8]. Targeting of linc01503/miR-4492/FOXK1 axis may serve as an effective target for CRC patients. The research directed by He et al. uncovered that patients with higher levels of linc01503 demonstrated inferior rates in survival condition in contrast to individuals exhibiting lower levels of linc01503 [28]. Linc01503 was determined to recruit the SFPQ to activate the transcription of FOSL1, thereby facilitating NPC cell proliferation, migration, and invasion [28]. The discovery of linc01503-induced pathway may introduce a prospective therapeutic target for patients with NPC. Interestingly, Li et al. uncovered the involvement of linc01503 in CBP resistance in OCa cells [17]. Mechanistic results showed that GATA1-activated LINC01503/miR-766-5p/PD-L1 network exerted an essential role in CBP resistance in OCa, illuminating a prospective target for improving the efficacy of OCa chemotherapy [17]. However, extensive studies are urgently needed to further the understanding of linc01503 associated drug resistance in various cancers.

## Conclusion and perspective

LncRNAs were initially thought to be functionless byproducts. The mystery of lncRNAs has gradually been unveiled owing to the implication of high-throughput screening technology. A variety of lncRNAs have been elucidated to regulate cancer occurrence and progression through various molecular mechanisms, such as acting as miRNA sponges, interacting with RBPs, and regulating expression of parental genes [30].

Despite much advances in the research of lncRNAs, there is still a long way ahead for lncRNAs to be incorporated into clinical practice. It is essential to develop a standard database of lncRNAs, fully revealing the origin, expression, function and current advances of lncRNA in different types of diseases, especially for malignant diseases. Further, larger tissue samples, longer follow-up visits, and the conduction of in vivo assays are proposed to unveil the identification of lncRNAs and improve the development of the molecular diagnosis. Furthermore, the stable expression of lncRNAs in blood plasma may pave a new path for cancer diagnosis and treatment. However, considerable work is needed to solve the difficulties and defects of lncRNA-based diagnosis and monitoring, such as high expense, existence of secondary structure, and limited knowledge of mechanism. Much more emphasis should be attached on the development of effective markers, which possess the characteristics of high sensitivity and specificity.

In this review, we are first reviewing the functional role, mechanistic models and clinical utilities of linc01503 in human cancers. In future, more tissue samples should be used to further determine the expression pattern of linc01503 in different cancers and further clarify the correlation among linc01503 level, clinicopathological characteristics and prognosis of cancer patients. Multiple effects between linc01503 and molecular targets should be explored in depth, thus facilitating the clinical implication. Furthermore, the expression pattern and molecular mechanism of linc01503 in body fluids are completely unknown, which are also needed.
